# The Characterization of G-Quadruplexes in Tobacco Genome and Their Function under Abiotic Stress

**DOI:** 10.3390/ijms25084331

**Published:** 2024-04-14

**Authors:** Kangkang Song, Bin Li, Haozhen Li, Rui Zhang, Xiaohua Zhang, Ruiwei Luan, Ying Liu, Long Yang

**Affiliations:** 1 College of Plant Protection and Agricultural Big-Data Research Center, Shandong Agricultural University, Tai’an 271018, China; kangkangsong234@163.com (K.S.); libin2994429664@163.com (B.L.);; 2 State Key Laboratory of Plant Physiology and Biochemistry, College of Life Sciences, Zhejiang University, Hangzhou 310058, China; 3 College of Agronomy, Shandong Agricultural University, Tai’an 271018, China

**Keywords:** tobacco, G-quadruplex, feature region, simple sequence repeat (SSR), abiotic stress

## Abstract

Tobacco is an ideal model plant in scientific research. G-quadruplex is a guanine-rich DNA structure, which regulates transcription and translation. In this study, the prevalence and potential function of G-quadruplexes in tobacco were systematically analyzed. In tobacco genomes, there were 2,924,271,002 G-quadruplexes in the nuclear genome, 430,597 in the mitochondrial genome, and 155,943 in the chloroplast genome. The density of the G-quadruplex in the organelle genome was higher than that in the nuclear genome. G-quadruplexes were abundant in the transcription regulatory region of the genome, and a difference in G-quadruplex density in two DNA strands was also observed. The promoter of 60.4% genes contained at least one G-quadruplex. Compared with up-regulated differentially expressed genes (DEGs), the G-quadruplex density in down-regulated DEGs was generally higher under drought stress and salt stress. The G-quadruplex formed by simple sequence repeat (SSR) and its flanking sequence in the promoter region of the *NtBBX* (*Nitab4.5_0002943g0010*) gene might enhance the drought tolerance of tobacco. This study lays a solid foundation for further research on G-quadruplex function in tobacco and other plants.

## 1. Introduction

**Figure 1:**
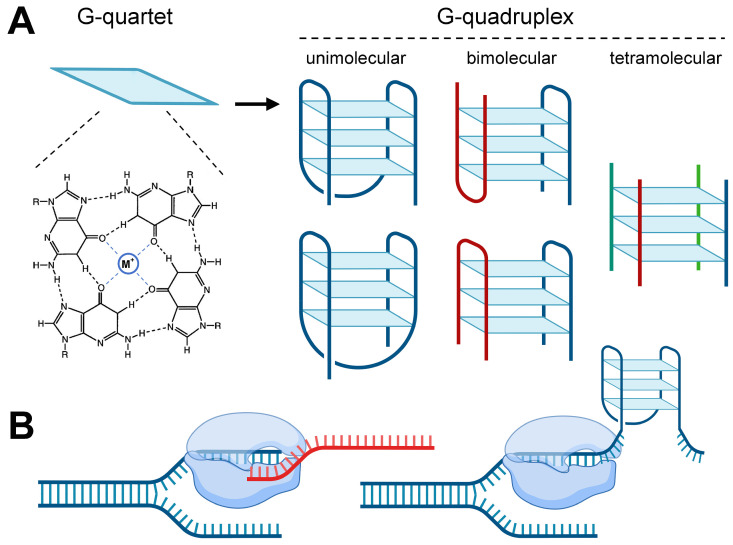
The structure and function of G-quadruplex. (**A**) G-quadruplex structure. Two or more G-quartets are stacked together to form the G-quadruplex structure, including unimolecular, bimolecular, and tetramolecular G-quadruplexes. (**B**) G-quadruplexes located on template strand generally inhibit transcription. The diagram was created with BioRender (https://www.biorender.com/ (accessed on 8 April 2024)).

A G-quadruplex is a nucleic acid secondary structure formed by folding nucleotide sequences rich in guanine bases in DNA and RNA [1]. Four guanines are connected to each other by Hoogsteen hydrogen bonds to form a G-quartet, and two or more G-quartets are stacked to shape a G-quadruplex structure (Figure 1A) [2]. Monovalent cations, such as K^+^ and Na^+^, are often bound to the central position of the G-quadruplex to enhance its stability [3]. According to the number of DNA strands forming a G-quadruplex structure, G-quadruplexes are divided into unimolecular, bimolecular, and tetramolecular G-quadruplexes [4]. In addition, the different number of continuous guanines in conservative motifs will lead to different G-quartet layers, so G-quadruplex can be divided into different types, of which G2 (with two G-quartets) and G3 (with three G-quartets) G-quadruplexes are the most common [5]. The most emblematical G-quadruplex sequence pattern is G_3+_N_1−7_G_3+_N_1−7_G_3+_N_1−7_G_3+_. A large number of studies have shown that the G-quadruplex plays an important regulatory role in DNA replication, transcription, translation, and telomere structure maintenance (Figure 1B) [1,6,7,8].

G-quadruplexes are widely prevalent in various plant genomes. In *Arabidopsis thaliana*, grape, rice, and *Populus tomentosa*, G-quadruplexes were usually located near transcription units or genes [9]. In *Arabidopsis thaliana* and rice, the content of G-quadruplexes at the 5’UTR/CDS junction was very rich [7]. Circular dichroism confirmed that a G-quadruplex structure can be formed in long terminal repeats in the maize LTR retrotransposon [10]. *Chlamydomonas reinhardtii* has a GC-rich genome (67%) and a high density of potential G-quadruplex formation sequences, which are located in the promoter region of DNA repair and photosynthetic genes [11]. A total of 23,685 G4 peaks were identified by chromatin immunoprecipitation of the BG4 antibody used to visualize the G-quadruplex structure and high-throughput sequencing in rice genome [12]. In *Spirodela polyrhiza*, there were strong G4 peaks in the promoters of the cytosolic nitrate reductase gene (*SpNR*) and nitrite reductase gene (*SpNiR*), which were located between −265 and −290 bp relative to the translation initiation site [13]. A stable G-quadruplex was revealed in the *RPB1* gene encoding the RNA polymerase II subunit by bioinformatics and circular dichroism [14]. G-quadruplexes were enriched in 5’UTR and 3’UTR of pea, suggesting the role of the G-quadruplex in post-transcriptional regulation [15]. In the barley genome, the G-quadruplex motif reached a peak near the 5’UTR, the first coding domain sequence and the first intron initiation site on the antisense strand [16]. The 5’UTR of ataxia telangiectasia mutated and rad3-related (ATR) mRNA in *Arabidopsis thaliana* was confirmed to have the G-quadruplex structure determined by biophysical and biochemical methods [17]. Using the nBMST computer program, the G-quadruplex structure was found to be enriched at the centromere of oat, and it may be formed in centromere duplication and the ENH3 nucleosome in vivo [18]. The prevalence and distribution of the G-quadruplex in the plant genome might vary with specific G-quadruplex types, and the G-quadruplex of G3 type was more abundant in intergenic regions, while the G-quadruplex of G2 type was established in gene regions [5].

The G-quadruplex plays a significant function in regulating the growth and development of plants. Notably, G-quadruplexes can influence gene expression via various mechanisms, both enhancing or suppressing it. In various plants, the conservative pattern of high-density G-quadruplexes at the promoter and 5’UTR positions indicated that G-quadruplexes have a vital role in regulating gene expression [19]. The G-quadruplex enriched in an intron may affect the transcription process [20]. The G-quadruplex in the 3’UTR region of *Arabidopsis* strongly enhanced the stability of mRNA and inhibited its degradation [21]. In *Sapium sebiferum*, the G-quadruplex at the L-ascorbate peroxidase gene may fulfill a crucial function in the flowering process [22]. G-quadruplexes were also common in genes related to energy steady-state signals and in many genes related to target of rapamycin (TOR), adenosine monophosphate (AMP) kinase, and the oxidative stress signal pathway, indicating that the G-quadruplex had an important action in energy state regulation, signal transduction, and metabolic regulation [23]. The G-quadruplex in long terminal repeats in the maize LTR retrotransposon inhibits the expression of the reporter gene in yeast [8]. The formation of a plant RNA G-quadruplex inhibited the translation of *SMXL4/5* and restricted phloem differentiation [24]. Genetic and biochemical analysis showed that RNA G-quadruplex folding can regulate translation and plant growth [25]. In *Spirodela polyrhiza*, the complex regulation of the nitrogen assimilation gene involved the synergistic effect of multiple NRElike and GAATC/GATTC cis-elements and TATA-based enhancers, (GA/CT)n repeats, and the G-quadruplex structure of promoters [13]. GO enrichment analysis has shown that an orthologous gene with a G-quadruplex in many dicotyledonous plant species is involved in important biological pathways, such as chromatin modification, the regulation of phosphorylation and intracellular signal transduction, auxin transport, seed development, and GTPase activity [16]. In monocotyledonous plant species, an orthologous gene with a G-quadruplex participates in biological processes, such as development, ion transport, transcriptional regulation, and protein folding [16].

G-quadruplexes are also involved in the process of plant response to abiotic stress. A G-quadruplex exists in the nuclease supersensitive site in the promoter of the rice thermal response gene. At simulated physiological temperature and potassium concentration, representative G-quadruplexes can form stable G-quadruplex structures, which can block DNA polymerase. However, with the increase in temperature, some G-quadruplexes disappear, which implies that these G-quadruplexes can sense temperature changes through structural transformation [26]. Plants growing in a low-temperature climate contained more guanine and G-quadruplexes in their transcriptome. Cold conditions were likely to strongly promote the folding of the RNA G-quadruplex with a higher number of G-quartets and medium loop length. GO analysis of the genes with a higher RNA G-quadruplex folding fraction after low-temperature treatment showed the enrichment of specific transcription in biological functions, such as the response to abiotic stimuli, response to temperature stimuli, and response to cold [25]. There were also many G-quadruplexes in maize hypoxia response genes, and the expression pattern of maize hypoxia response genes carrying G-quadruplexes could be changed under the supply of sugar [19,27]. G-quadruplexes were also found to be enriched in differentially expressed genes of *Arabidopsis thaliana* under drought stress [28].

Tobacco is widely used in scientific research and is also an important cash crop. The G-quadruplex is an important DNA structure, which is widely involved in key life processes. However, unlike humans and animals, the research on the G-quadruplex in plants is very limited. With the release of high-quality genomes and the development of bioinformatics, the G-quadruplex has been systematically studied in *Arabidopsis thaliana* [9,28], rice [12,29], wheat [30], barley [16], and pea [15]. In this study, the G-quadruplex in tobacco was systematically analyzed by the bioinformatics method, using tobacco genome data and transcriptome data under abiotic stress. The G-quadruplex of feature sequences, the relationship between simple sequence repeats (SSRs) and the G-quadruplex, the G-quadruplex of differentially expressed genes (DEGs), and the G-quadruplex of the transcription factor gene family were carefully investigated. This study promotes the understanding of the prevalence and function of the G-quadruplex in tobacco and other plants.

## 2. Results

### 2.1. General Situation of G-Quadruplex Distribution in Tobacco Genome

The length of tobacco chromosomes ranged from 82,751,733 bp (Nt21) to 215,930,317 bp (Nt17). The GC content was between 38.6% (Nt19) and 39.5% (Nt01). The differences in the number of G-quadruplexes in chromosomes were large, with the smallest being 42,881 (Nt11) and the largest being 116,804 (Nt17) (Table 1). The density of G-quadruplexes in all nuclear chromosomes was basically the same, with an average density of 0.5/kbp. The density of G-quadruplexes in the mitochondrial chromosome was 1.6/kbp, and the density of G-quadruplexes in the chloroplast chromosome was 0.9/kbp.

The potential G-quadruplex forming sequences in the nuclear genome amount to 2,924,271,002. The most abundant G-quadruplex sequence, “GGGGGTGTGTACAGACTCCGGAGGGG”, occurred 1302 times in the genome. The other four most abundant G-quadruplex sequences occurred approximately 600 times in the genome. These sequences had a roughly equal probability of occurrence on both the positive and negative strands (Table 2).

### 2.2. The Relationship between Tobacco G-Quadruplexes and Genome Characteristics

**Figure 2:**
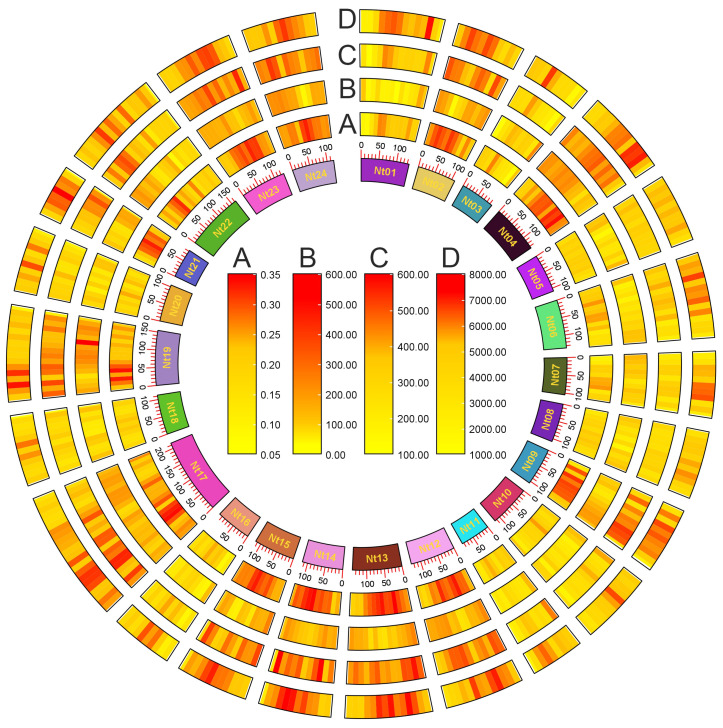
G-quadruplex landscape in tobacco genome. (A) GC content. (B) Gene density. (C) SSR density. (D) G-quadruplex density. The innermost circle represents the 24 chromosomes of tobacco. Chromosomes are divided into bins of 10 Mb each.

The prevalence of tobacco G-quadruplexes was potentially influenced by GC content, gene density, and SSR density (Figure 2). In some genome regions, such as 30–40 Mb of the Nt03 chromosome and 40–50 Mb of the Nt20 chromosome, GC density, gene density, SSR density, and G-quadruplex density collectively exhibited higher levels compared to neighboring DNA regions. The effect of SSRs on G-quadruplex formation was investigated emphatically. A total of 109,865 SSRs were identified in the tobacco genome, among which p2 SSRs were largest, accounting for 51.95% of all SSRs, followed by p3 SSRs, accounting for 33.68% of all SSRs (Figure S1). Among p3 SSRs, the number of SSRs with repeating units of CCN, NGG, NCC, GGN, CNC, and GNG were 275, 400, 689, 340, 272, and 471 respectively, and these 2447 SSRs account for 6.61% of p3 SSRs and 2.23% of all SSRs. There are 34,553 p3 SSRs with AAN, NTT, NAA, TTN, TNT, ANA, and other types, accounting for 93.39% of p3 SSRs and 31.45% of all SSRs (Figure S1). A total of 5906 SSRs could form 1–15 G-quadruplexes, including 1692 p3 SSRs. A 132 bp SSR on the Nt07 chromosome, (AAG)5attttgg(ATA)5(aga)6ggttggata(agg)8at(gag)8a(agg)5, had the potential to form 15 G-quadruplexes (Table S1). SSRs in the tobacco genome could form a total of 7679 G-quadruplexes. These results implied that SSRs may indirectly affect the distribution of G-quadruplexes by impacting GC density and gene density (Figure 2).

### 2.3. G-Quadruplex of Feature Regions in Tobacco Genome

The number of G-quadruplexes varied greatly across different genomic feature regions (Table S2). The number of G-quadruplexes in intergenic regions reached as high as 2,954,439, while only 484 were present in the 3’UTR region. Among the various regions of genes, introns harbored the highest number of G-quadruplexes, while the exons, CDS, and 5’UTR contained fewer, with the 3’UTR hosting the least. A large number of G-quadruplexes were also found in promoters and TSS500 regions. Additionally, the number of G-quadruplexes varied between different DNA strands within the same genomic feature region. In certain regions such as the 5’UTR, there is a notable difference in the quantity of G-quadruplexes between the template and coding strands.

**Figure 3:**
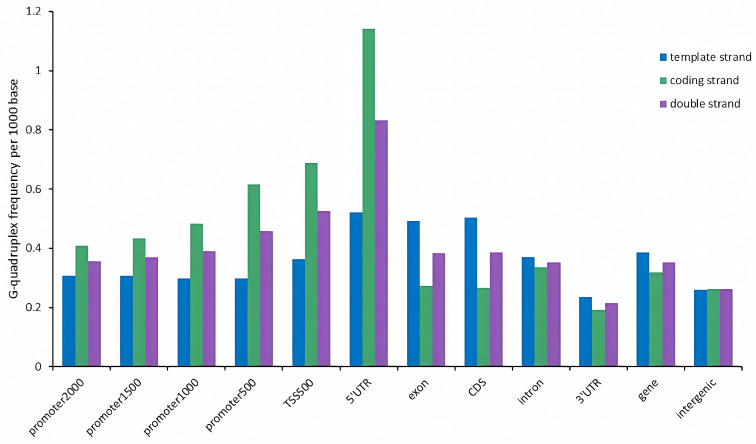
The density of G-quadruplexes in different feature regions of tobacco genome. The promoter2000, promoter1500, promoter1000, and promoter500 represent the sequences upstream of the gene by 2000 bp, 1500 bp, 1000 bp, and 500 bp, respectively. TSS500 represents the sequence consisting of 250 bp upstream and downstream of the transcription start site.

The density of G-quadruplexes in different genomic feature regions was different. In specific feature regions, the density of G-quadruplexes on template strands and coding strands was also different (Figure 3). On the DNA double strands, the G-quadruplex density in genomic feature regions was as follows, from highest to lowest: 5’UTR, TSS500, promoter500, promoter1000, CDS, exon, promoter1500, promoter2000, intron, gene, intergenic, and 3’UTR. In three sequence contexts (double strand, template strand, and coding strand), the G-quadruplex density was highest in the 5’UTR region and lowest in the 3’UTR region. For the promoter region, the G-quadruplex density follows the same pattern on both the double strand and coding strand, whereby shorter promoter sequences correspond to higher G-quadruplex density. Conversely, the G-quadruplex density in promoter sequences on the template strand exhibited an opposite trend, with shorter promoter sequences exhibiting lower G-quadruplex density.

### 2.4. Functions of Genes with Highly Enriched G-Quadruplexes in Promoters

**Figure 4:**
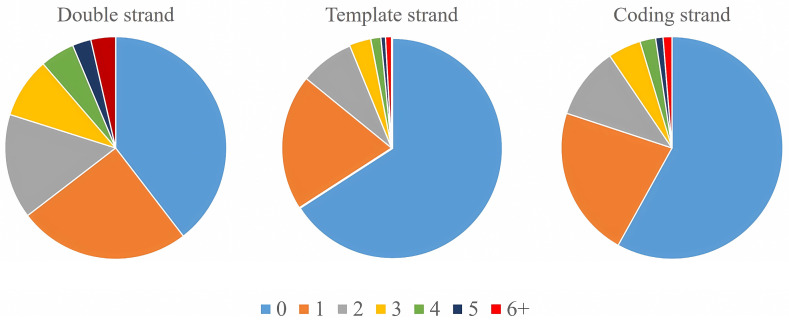
The proportion of genes with different G-quadruplex numbers in the promoter. The colors indicate genes containing various G-quadruplex numbers in the promoter. The value of 0 represents genes whose promoter missed G-quadruplexes. The values of 1, 2, 3, 4, and 5 represent genes whose promoter contains one, two, three, four, and five G-quadruplexes, respectively. The value of 6+ represents the genes whose promoter contains six or more G-quadruplexes.

G-quadruplexes were enriched in different numbers in promoters of genomic genes, and there were also differences among the three sequence contexts (double strand, template strand, and coding strand) (Figure 4). In the template strand, G-quadruplexes were absent in the promoter regions of 23,401 genes, accounting for 65.88% of all genes in the genome. In the promoter regions of 11,806 genes, there were 1–5 G-quadruplexes, accounting for 33.24% of all genes. The promoter regions of 312 genes were capable of forming six or more G-quadruplexes, representing 0.88% of all genes.

**Figure 5:**
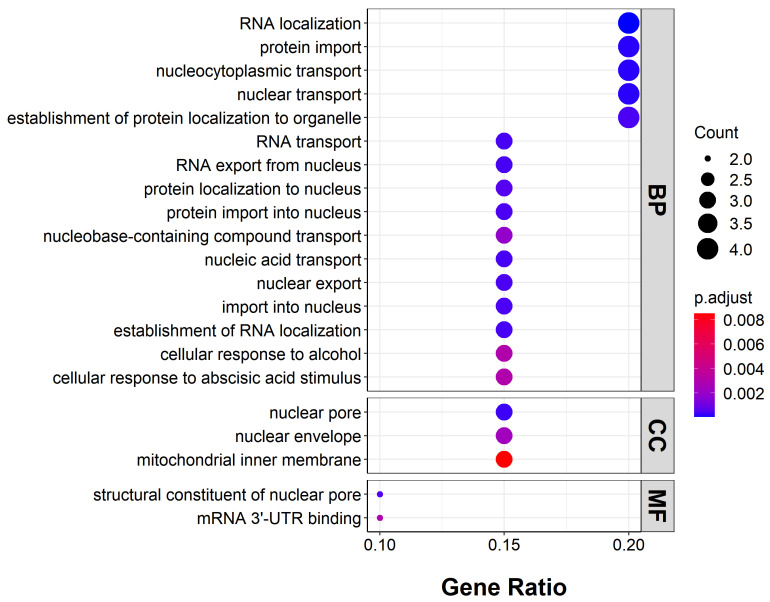
GO enrichment of genes rich in promoter G-quadruplex (>10) in template strand.

The genes with more than 10 G-quadruplexes in the promoter template strand were selected for GO function enrichment analysis. The enrichment of BP (biological process), CC (cellular component), and MF (molecular function) ontologies was calculated and 21 enriched terms were found (Figure 5). The biological process category included RNA localization, protein import, nucleocytoplasmic transport, nuclear transport, the establishment of protein localization to organelles, RNA transport, RNA export from nuclei, protein localization to nuclei, protein import into nuclei, nucleobase-containing compound transport, nucleic acid transport, nuclear export, import into nuclei, the establishment of RNA localization, the cellular response to alcohol, and the cellular response to abscisic acid stimuli. The cellular component category included nuclear pores, nuclear envelopes, and mitochondrial inner membranes. The molecular function category included structural constituents of nuclear pores and mRNA 3’UTR binding. These aspects involve the important process of plant growth and development.

### 2.5. G-Quadruplex of DEGs under Abiotic Stress

**Figure 6:**
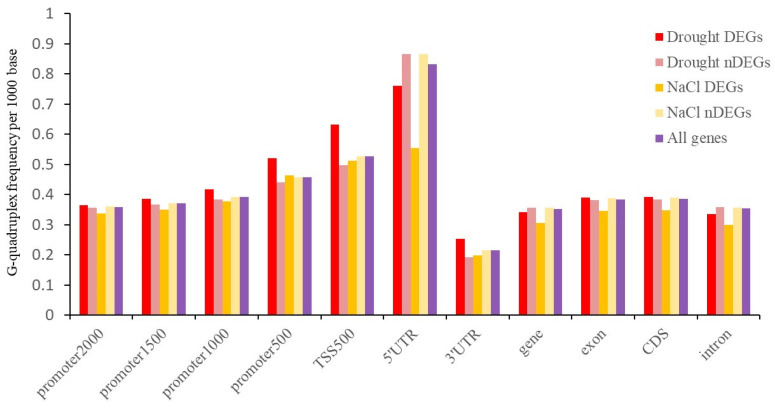
The density of G-quadruplex of DEGs and nDEGs under drought stress and salt stress.

Under drought stress, 7477 genes were differentially expressed (2718 up-regulated DEGs and 4759 down-regulated DEGs). Under salt stress, 2764 genes were differentially expressed (1198 up-regulated DEGs and 1566 down-regulated DEGs) (Table S3). Comparing DEGs and non-differentially expressed genes (nDEGs) under drought stress and salt stress, the number and density of G-quadruplexes in different feature regions showed distinct characteristics (Table S4, Figure 6). For the G-quadruplex density of the gene, intron, and 5’UTR regions, DEGs were lower than nDEGs. This pattern was especially obvious in the 5’UTR region under NaCl stress. For the G-quadruplex density of the CDS, 3’UTR, promoter2000, promoter1500, promoter1000, and TSS500, DEGs were higher than nDEGs under drought stress, but it exhibited the opposite trend under NaCl stress. Notably, for the G-quadruplex density of each feature region, DEGs of drought stress were higher than DEGs of salt stress.

**Figure 7:**
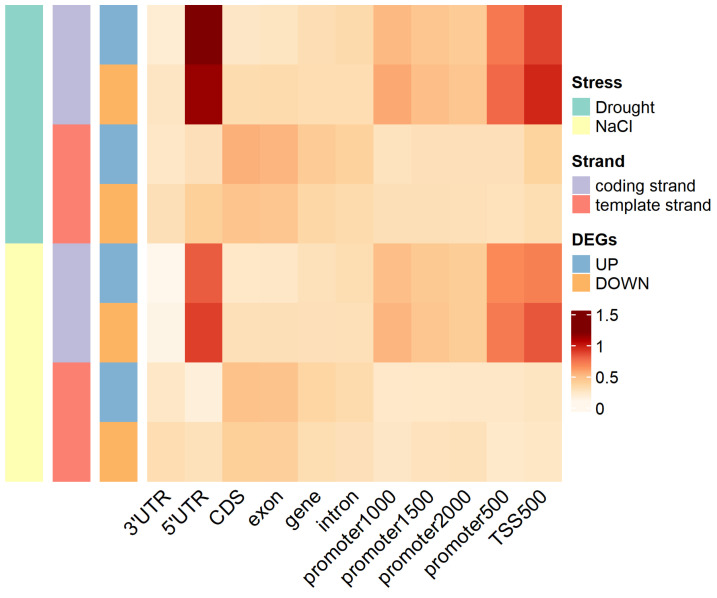
The density of G-quadruplexes in different feature regions of DEGs under drought stress and NaCl stress.

G-quadruplexes of up-regulated DEGs and down-regulated DEGs were investigated in further detail. For up-regulated DEGs and down-regulated DEGs under drought stress and salt stress, the number and density of G-quadruplexes in different feature regions were different, and the template strand and coding strand in specific feature regions were also different (Tables S5 and S6 and Figure 7). Except for the template strand in the 3’UTR region, the density of G-quadruplexes of DEGs under drought stress was higher than that under salt stress in all other cases (Figure 7).

Whether up-regulated DEGs or down-regulated DEGs under drought stress, for the G-quadruplex density of the 5’UTR, TSS500, and promoter regions, the coding strand was higher than the template strand. In up-regulated DEGs under drought stress, for the G-quadruplex density of the 5’UTR region, the coding strand was 4.3-fold higher than the template strand. In down-regulated DEGs under drought stress, for the G-quadruplex density of the 5’UTR region, this value was 2.9-fold higher. In addition, whether up-regulated DEGs or down-regulated DEGs under drought stress, for the G-quadruplex density of the 3’UTR, gene, exon, CDS, and intron regions, the coding strand was lower than the template strand. In up-regulated DEGs under drought stress, for the G-quadruplex density of the CDS region, the coding strand in the CDS region was 2.2-fold lower than the template strand. In down-regulated DEGs under drought stress, for the G-quadruplex density of the CDS region, this value was 1.4-fold lower.

This strong model was also revealed under NaCl stress. In up-regulated DEGs under NaCl stress, for the G-quadruplex density of the 5’UTR region, the coding strand was 5.1-fold higher than the template strand. In down-regulated DEGs under NaCl stress, for the G-quadruplex density of the TSS500 region, this value was 3.5-fold higher. In addition, in up-regulated DEGs under NaCl stress, for the G-quadruplex density of the 3’UTR region, the coding strand was 2.4-folds lower than the template strand. In down-regulated DEGs under NaCl stress, for the G-quadruplex density of the 3’UTR region, this value was 2.6-fold lower.

### 2.6. G-Quadruplexes in Transcription Factor Gene Family

**Figure 8:**
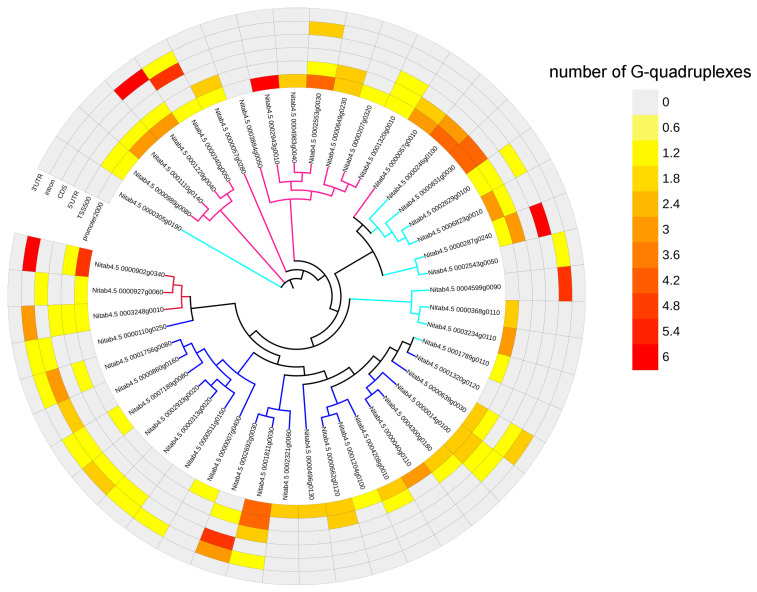
G-quadruplex in four transcription factor gene families responding to drought stress. The gene family types are represented by the color of the branches of the phylogenetic tree, with cyan as *bZIP* gene family members, blue as *NAC* gene family members, deep pink as *BBX* gene family members, and red as *MADS-box* gene family members. The color of the heat map represents the number of G-quadruplexes. The phylogenetic tree was constructed using maximum likelihood.

The bZIP genes, NAC genes, BBX genes, and MADS-box genes were differentially expressed under drought stress, and most of these DEGs contained G-quadruplexes in their gene body and transcriptional regulatory regions (Figure 8). The promoter region of some genes comprised multiple G-quadruplexes, such as *Nitab4.5_0000246g0100* (*bZIP*, 4), *Nitab4.5_0000831g0030* (*bZIP*, 4), *Nitab4.5_0001811g0030 (NAC*, 4), *Nitab4.5_0002943g0010* (*BBX*, 6), and *Nitab4.5_0000902g0340* (*MADS-box*, 5).

**Figure 9:**
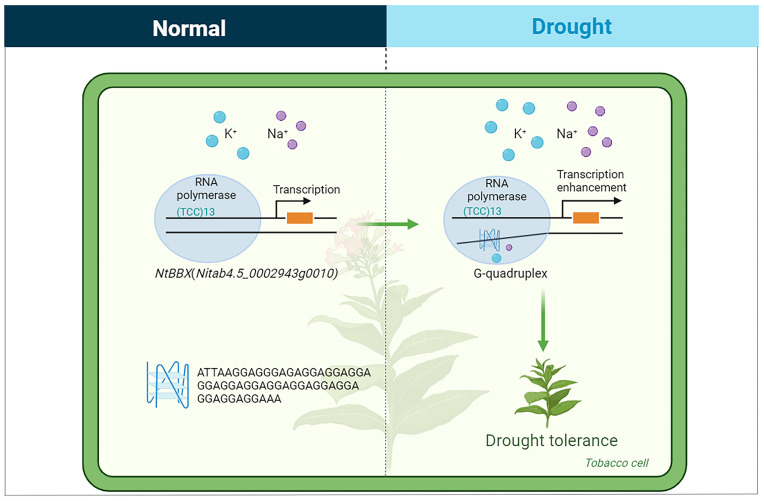
The G-quadruplex formed by SSR and its flanking sequence in the promoter region of the *NtBBX* (*Nitab4.5_0002943g0010*) gene might enhance drought tolerance in tobacco. The drawing was created with BioRender (https://www.biorender.com/ (accessed on 15 December 2023)).

SSRs were involved in the formation of a G-quadruplex in the gene expression regulatory region of two transcription factors, namely *Nitab4.5_0002692g0030* and *Nitab4.5_0002943g0010*. *Nitab4.5_0002692g0030* belonged to the *NAC* gene family, which was up-regulated under drought stress. In the second intron region of this gene, an SSR with (GGA)5 engaged the formation of a G-quadruplex. *Nitab4.5_0002943g0010* belonged to the *BBX* gene family, which was up-regulated under drought stress. In the promoter region of this gene, an SSR participated in the formation of a G-quadruplex, and the repeat unit of this SSR was GGA, with 13 repeats (Figure 9).

## 3. Discussion

### 3.1. Tobacco Organelle Genome Has Higher G-Quadruplex Density than Nuclear Genome

The density of G-quadruplexes in tobacco nuclear DNA was 0.5/kbp, while those in mitochondrial DNA and chloroplast DNA were 1.6/kbp and 0.9/kbp, respectively. A recent study on G-quadruplexes of pea confirmed the conclusion that the organelle genome had higher G-quadruplex density [15]. It is worth noting that the GC content of the tobacco mitochondrial genome is not significantly higher than that of the nuclear genome, and the GC content of the chloroplast genome is even lower than that of the nuclear genome. Therefore, GC content is not the only factor that causes the difference in G-quadruplex density between the organelle genome and the nuclear genome.

In the study of non-plant species, the mitochondrial genome G-quadruplex has been proved to play a direct role in mitochondrial genome replication, transcription, and respiratory function [31]. However, the function of these special advanced DNA structures in plant mitochondria is still unclear. The tendency of the G-quadruplex to be enriched in the genome of tobacco organelles suggests that the G-quadruplex plays a specific role in some physiological and biochemical processes. Outside the nucleus, there are important genomes in mitochondria and chloroplasts, and these extranuclear genes play a vital role in respiration, photosynthesis, and development [32,33]. At present, the editing schemes of the tobacco mitochondrial genome and chloroplast genome have been put forward [34,35]. In the future, gene editing technology can be used to change the base arrangement in the motif of the G-quadruplex in tobacco organelles to control the formation and stability of the G-quadruplex, to change the expression level of related genes and ultimately change the yield and quality of tobacco.

### 3.2. G-Quadruplex Was Enriched in the Coding Strand of Upstream Regulatory Region

The density of the G-quadruplex in the tobacco promoter, TSS500, and 5’UTR regions is higher than that in other genomic feature regions, and the density of the G-quadruplex in the coding strand of these three feature regions is obviously higher than that of the template strand. The promoter, TSS500, and 5’UTR regions all belong to the upstream regulatory regions of the gene, and the enrichment of the G-quadruplex in these regions suggests that they may play an important role in regulating gene expression.

Whether these G-quadruplex structures in the upstream regulatory region of tobacco genes promote or inhibit gene expression needs further exploration. The G-quadruplex has long been considered an obstacle to gene expression. In 2012, the concept of the G-quadruplex as a direct transcription repressor was challenged [36]. Subsequently, more and more studies show that the G-quadruplex does not have a simple inhibitory effect on gene expression regulation, and many studies have found the relationship between the G-quadruplex and high transcription activity [37,38,39]. In rice, the G-quadruplex in the gene body was negatively correlated with gene expression, while when the G-quadruplex was located in the promoter, it was positively correlated with gene expression [12]. The G-quadruplex in the tobacco genome may also play a dual role in tobacco gene expression. The study on the location of the G-quadruplex in the tobacco genome and the effect of its quantity on gene expression activity will be helpful in the application of the G-quadruplex in improving tobacco characteristics.

### 3.3. SSR Provided Conditions for G-Quadruplex Formation

The SSR is widely distributed in the tobacco genome, and its high variability and relatively conservative flanking sequences provide conditions for the formation of G-quadruplexes. In tobacco, SSRs forming G-quadruplexes account for 5.38% of all SSRs, and the proportion is not high. This is determined by the formation of G-quadruplexes, which requires a high G content in the sequence and the specific algorithm logic of the G4Hunter program. Among all SSRs in the tobacco genome, p2 SSRs have the largest number, accounting for 51.95% of all SSRs, and the actual proportion is even larger than this value, because the compound SSR also contains dinucleotide repeats, which is similar to wheat [40] and rice [41]. However, all p2 SSR types have no potential to form a G-quadruplex. The number of p3 SSRs is also large, accounting for 33.68% of all SSRs. In p3 SSRs, only SSRs with repeating units of CCN, NGG, NCC, GGN, CNC, and GNG can form G-quadruplexes. However, in p3 SSRs of tobacco, the proportion of AAN, NTT, NAA, TTN, TNT, ANA, and other types of repetitive motifs is high, and p3 SSRs with G-quadruplex formation potential only account for 6.61% of all p3 SSRs. p2 SSRs and p3 SSRs account for a very high proportion in the genome, but few of them have the potential to form G-quadruplexes, which is the main reason for the small number of SSRs involved in the formation of G-quadruplexes.

In tobacco, although the SSR involved in the formation of G-quadruplexes only accounts for a small part of all SSRs in the genome, there are 5906 SSRs capable of forming G-quadruplexes, which is very considerable. The association between a large number of SSRs and G-quadruplexes in tobacco suggests that they may have the function of regulating gene expression. The appearance of these structures increases the instability of genes and may affect the process of gene expression, including DNA replication, repair, and transcription. In specific areas of the tea genome, a correlation between SSR density and the G-quadruplex, GC density, gene density, and CRISPR editing site density was found, and these areas were related to the secondary metabolism of tea [42]. In addition to SSRs, there are other types of tandem repeats and scattered repeats in the plant genome, including satellite DNA and transposons. The relationship between these repeats and the G-quadruplex in the tobacco genome needs further study.

### 3.4. G-Quadruplex May Be Involved in the Response of Abiotic Stress in Tobacco

Abiotic stress has a significant impact on plant growth and development. The transcription factor is the main regulator of abiotic stress and an excellent candidate gene for crop improvement [43]. The *bZIP*, *NAC*, *BBX*, and *MADS-box* genes, as important transcription factors, have been found to perform crucial functions in tobacco growth and abiotic stress tolerance [44,45,46,47]. There are a large number of G-quadruplexes in the feature region of tobacco DEGs under salt stress and drought stress, among which four transcription factor family genes, *bZIP*, *NAC*, *BBX*, and *MADS-box*, are expressed in different degrees under drought stress. During abiotic stress such as drought, the cytoplasmic concentration of K^+^ and Na^+^ cations increased [48]. It is known that higher levels of K^+^ and Na^+^ can promote the formation of a G-quadruplex [3], and the ability of the G-quadruplex motif to form a G-quadruplex structure may change under drought stress and salt stress, thus affecting the transcription activity of genes to varying degrees. Therefore, the dynamic changes of G-quadruplex stability in the DEG promoter under drought stress and salt stress may be the potential mechanism for tobacco to cope with these two abiotic stresses. Studies in rice have proved that the G-quadruplex on the promoter can promote gene expression [12]. The G-quadruplex in the upstream regulatory region of the tobacco gene may also promote gene expression. A possible model of the *NtBBX* transcription factor (*Nitab4.5_0002943g0010*) gene in drought tolerance was proposed. The *NtBBX* transcription factor (*Nitab4.5_0002943g0010*) is an up-regulated differentially expressed gene under drought stress, and its promoter coding strand has a p3 SSR, which has the ability to form a G-quadruplex with its flanking sequence. It is known that K^+^ and Na^+^ can increase the stability of a G-quadruplex, and the increase in intracellular K^+^ and Na^+^ concentration under drought stress can promote the formation of a G-quadruplex, and then promote the expression of the *NtBBX* transcription factor to enhance the drought stress tolerance of tobacco (Figure 9). It should be emphasized that this hypothesis only preliminarily revealed that the G-quadruplex induced by SSR regulated the expression of the transcription factor. This potential relationship provides an important direction for future research, and the applicability of this hypothesis to all genomic genes needs to be explored from multiple perspectives in future work. Under certain conditions such as abiotic stress, whether the DNA G-quadruplexes in root, shoot, and flower parts are different is also an interesting and important direction for future research. For the key G-quadruplex that regulates plant development and environmental adaptation, the nucleotide that constitutes the G-quadruplex can be mutated by gene editing technology to control the formation and stability of the G-quadruplex, to enhance the resistance of cash crops such as tobacco to abiotic stress.

## 4. Materials and Methods

### 4.1. In Silico Identification and Characterization of G-Quadruplexes in Tobacco Genome

The tobacco nuclear genome was obtained from the Sol Genomics Network (https://solgenomics.net/ftp/genomes/Nicotiana_tabacum/edwards_et_al_2017/ (accessed on 18 August 2022)) [49]. The organelle genomes (mitochondrial genome, BA000042.1; chloroplast genome, Z00044.2) were acquired from the National Center for Biotechnology Information [50]. The nucleotide sequence of each chromosome was submitted to G4Hunter to identify the potential G-quadruplexes, with the window set to 25 and the threshold set to 1.2 [51]. The genome coordinates of each G-quadruplex were obtained. The G4Hunter data were further cleaned. The number and density of G-quadruplexes of each chromosome and detailed information of all G-quadruplexes, as well as the sequence length and GC content, were summarized. The SSRs in the tobacco genome were identified by MISA v2.1, which was set as default parameters (1-20 2-6 3-5 4-5 5-5 6-5, interruptions: 100, GFF: true) [52]. The GC density, gene density, SSR density, and G-quadruplex density of each chromosome were calculated and visualized using the Advanced Circos function module of Tbtools v1.108 [53].

According to the genome annotation file, the genome coordinates of feature regions, including the exon, CDS, intron, 5’UTR, 3’UTR, gene, intergenic, promoter2000, promoter1500, promoter1000, promoter500, and TSS500, were extracted and calculated. The promoter2000, promoter1500, promoter1000, and promoter500 were defined as the sequences upstream of the gene by 2000 bp, 1500 bp, 1000 bp, and 500 bp, respectively. TSS500 was defined as the sequence consisting of 250 bp upstream and downstream of the transcription start site. Based on the genome coordinates of the G-quadruplex and the genome coordinates of the above feature regions, the number of G-quadruplexes in each feature region was calculated, and the further density of G-quadruplexes in each feature region was calculated. These computations were all conducted using a customized R script.

### 4.2. Number Level of G-Quadruplexes in Promoter and Functional Enrichment Analysis

All genes of the tobacco genome were divided into seven classes based on the number of G-quadruplexes in the promoter region. The first class was the genes whose promoter missed G-quadruplexes. The second to sixth classes were genes with one, two, three, four, and five G-quadruplexes in their promoters, respectively. The seventh class was the genes whose promoter contained six or more G-quadruplexes.

Genes with more than 10 G-quadruplexes in the promoter template strand were selected as a set for Gene ontology (GO) enrichment analysis to explore which biological functions these G-quadruplex-enriched genes were related to. The functional annotation of these genes was performed through eggNOG-mapper (http://eggnog-mapper.embl.de/ (accessed on 19 October 2022)) by submitting the corresponding protein sequences [54]. The annotation result was sorted using the eggNOG-mapper Helper module of TBtools v1.108. GO enrichment analysis and visualization were performed through the clusterProfiler 4.0 R package [55].

### 4.3. G-Quadruplex Analysis of DEGs under Abiotic Stresses

RNA-seq data were obtained from the Sequence Read Archive (SRA) database (https://www.ncbi.nlm.nih.gov/sra/ (accessed on 21 December 2022)) [56], including drought stress (SRP301492) and NaCl stress (SRP193166). Trimmomatic v0.39 [57] was employed to remove adapters and cut off the first 12 bases of reads. The genome index was established and reads were mapped using Hisat2 v2.2.1 [58]. The sam files were converted to bam files by Samtools v1.7 [59]. The FPKM values were calculated by Stringtie v2.1.7 [60]. The counts values were obtained by the prepDE.py3 program provided by Stringtie. The DEGs were analyzed by using DEseq2 [61], where the screening standard was |log2FoldChange| ≥ 1 and padj ≤ 0.05. The gene IDs of DEGs, nDEGs, up-regulated DEGs, and down-regulated DEGs were gained. Then, the genome coordinates of all feature regions of these genes were obtained from the results in Section 4.1. Based on the genome coordinates of the G-quadruplex and genome coordinates of these genes, the number of G-quadruplexes in these genes was calculated, and the further density of G-quadruplexes in each feature region was calculated.

### 4.4. G-Quadruplex Analysis of Transcription Factor Genes Responding to Drought Stress

Annotated *bZIP*, *NAC*, *BBX*, and *MADS-box* family genes were extracted, and these genes were intersected with DEGs under drought stress to obtain the DEGs of these four transcription factor family genes under drought stress using the intersect R function. Based on the genome coordinates of the G-quadruplex and genome coordinates of these transcription factor genes, the number of G-quadruplexes in each transcription factor gene was calculated. The ML phylogenetic tree was constructed using the family protein sequences through the One Step Build a ML Tree function module of TBtools v1.108. The number of G-quadruplexes in differentially expressed family genes was displayed by iTOL (https://itol.embl.de/ (accessed on 24 March 2023)) [62].

## 5. Conclusions

In this study, a large number of G-quadruplexes were revealed to exist in the tobacco genome, and the G quadruplex density in the organelle genome was greater than that in the nuclear genome. G-quadruplexes were abundant in the regulatory region related to gene expression, and there are differences in G-quadruplex density between the coding strand and template strand. For the G-quadruplex density of DEGs under drought stress and salt stress, the down-regulated DEGs are generally higher than the up-regulated DEGs. The G-quadruplex formed by SSRs and the flanking sequences of the transcription factor promoter region might enhance the drought tolerance of tobacco. This study greatly promotes the understanding of the distribution and function of G-quadruplexes in tobacco and other plants and provides plentiful available genetic resources for future research.

## Figures and Tables

**Table 1:** G-quadruplex profile of tobacco genome. The G-quadruplexes were identified by G4Hunter, with the window set to 25 and the threshold set to 1.2.

Chromosome	Length (bp)	GC Content (%)	G-Quadruplex Number	G-Quadruplex Density (per kbp)
Nt01	135,559,120	39.5	69,547	0.5
Nt02	109,624,155	38.7	60,928	0.6
Nt03	97,104,660	39.1	43,104	0.4
Nt04	136,037,944	38.8	75,885	0.6
Nt05	109,337,480	39.3	53,838	0.5
Nt06	136,518,381	39.3	70,455	0.5
Nt07	105,049,242	39.2	51,317	0.5
Nt08	108,393,918	39.4	52,757	0.5
Nt09	106,147,314	38.8	57,485	0.5
Nt10	116,194,611	39.1	55,884	0.5
Nt11	84,914,900	39.2	42,881	0.5
Nt12	127,111,110	38.8	71,171	0.6
Nt13	139,740,185	38.8	82,542	0.6
Nt14	115,579,444	38.8	69,329	0.6
Nt15	115,424,069	39	66,956	0.6
Nt16	99,759,613	39.2	45,931	0.5
Nt17	215,930,317	38.8	116,804	0.5
Nt18	113,077,399	39	51,195	0.5
Nt19	155,028,365	38.6	81,044	0.5
Nt20	105,109,812	39.2	52,074	0.5
Nt21	82,751,733	39	47,122	0.6
Nt22	163,185,734	39	85,914	0.5
Nt23	128,150,892	38.7	71,924	0.6
Nt24	118,540,604	38.7	66,729	0.6
mitochondrion	430,597	45	705	1.6
chloroplast	155,943	37.8	148	0.9

**Table 2:** The five most frequent G-quadruplex motif families in the tobacco nuclear genome.

Sequence	Number	Positive Strand	Negative Strand	Length (bp)	ABS_Score
GGGGGTGTGTACAGACTCCGGAGGGG	1302	635	667	26	1.423077
GGGGGCCTCGGGTGTGTTTCGGATG	605	294	311	25	1.200000
GGGGTGTGTACAGACTCCGGAGGGG	587	306	281	25	1.320000
CGGGGGGTTGACTTTTTGATATCGGGGT	599	304	295	28	1.357143
CTGGGGGTGTACAGACTCCGGAGGGGCT	575	283	292	28	1.214286

## Data Availability

Data are contained within the article or Supplementary Materials. All R scripts have been submitted to GitHub (https://github.com/KangkangSong123/G4GenomeMAP/tree/master).

## Supplementary Materials

The following supporting information can be downloaded at https://www.mdpi.com/article/10.3390/ijms25084331/s1.
