# Supplementary material for: The Characterization of G-Quadruplexes in Tobacco Genome and Their Function under Abiotic Stress
Source: Int J Mol Sci. 2024 Apr 14;25(8):4331. doi: 10.3390/ijms25084331 (PMC11050182; doi:10.3390/ijms25084331)
Supplement: Supplementary file 1 [file ijms-25-04331-s001.zip › Supplementary Figure.pdf]

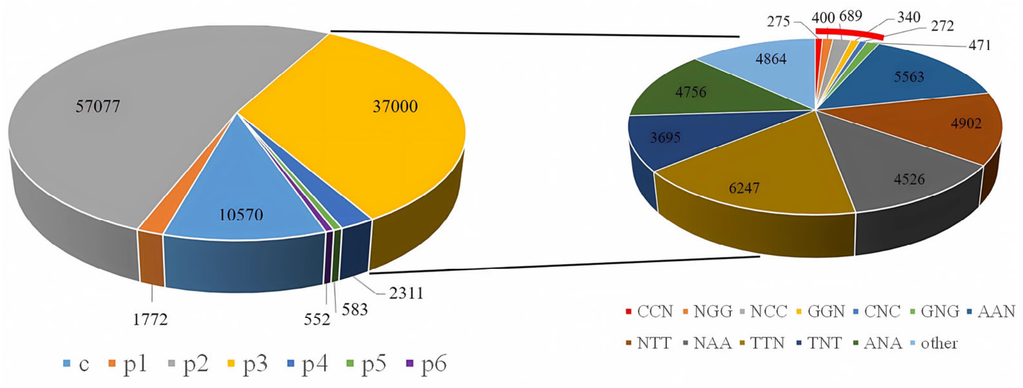

Figure S1. The type and number of SSRs in tobacco genome and the repeat unit category of p3 SSR. The p1, p2, p3, p4, p5, p6 represent SSR of triple base repeat single base repeat SSR, two base repeat SSR, triple base repeat SSR, four base repeat SSR, five base repeat SSR, six base repeat SSR, respectively. The c represents compound SSR.
